# Anisotropic Rashba splitting in Pt-based Janus monolayers PtXY (X,Y = S, Se, or Te)†

**DOI:** 10.1039/d1na00334h

**Published:** 2021-09-14

**Authors:** Paul Albert L. Sino, Liang-Ying Feng, Rovi Angelo B. Villaos, Harvey N. Cruzado, Zhi-Quan Huang, Chia-Hsiu Hsu, Feng-Chuan Chuang

**Affiliations:** Department of Physics, National Sun Yat-sen University 70 Lienhai Rd. Kaohsiung 80424 Taiwan fchuang@mail.nsysu.edu.tw +886-7-5253733; Institute of Mathematical Sciences and Physics, College of Arts and Sciences, University of the Philippines Los Baños, College Laguna 4031 Philippines; Physics Division, National Center for Theoretical Sciences Taipei 10617 Taiwan; Department of Physics, National Tsing Hua University Hsinchu 30013 Taiwan

## Abstract

Recent studies have demonstrated the feasibility of synthesizing two-dimensional (2D) Janus materials which possess intrinsic structural asymmetry. Hence, we performed a systematic first-principles study of 2D Janus transition metal dichalcogenide (TMD) monolayers based on PtXY (X,Y = S, Se, or Te). Our calculated formation energies show that these monolayer Janus structures retain the 1T phase. Furthermore, phonon spectral calculations confirm that these Janus TMD monolayers are thermodynamically stable. We found that PtSSe, PtSTe, and PtSeTe exhibit an insulating phase with indirect band gaps of 2.108, 1.335, and 1.221 eV, respectively, from hybrid functional calculations. Due to the breaking of centrosymmetry in the crystal structure, the spin–orbit coupling (SOC)-induced anisotropic Rashba splitting is observed around the M point. The calculated Rashba strengths from M to Γ (*α*^M–Γ^_R_) are 1.654, 1.103, and 0.435 eV Å^−1^, while the calculated values from M to K (*α*^M–K^_R_) are 1.333, 1.244, and 0.746 eV Å^−1^, respectively, for PtSSe, PtSTe, and PtSeTe. Interestingly, the spin textures reveal that the spin-splitting is mainly attributed to the Rashba effect. However, a Dresselhaus-like contribution also plays a secondary role. Finally, we found that the band gaps and the strength of the Rashba effect can be further tuned through biaxial strain. Our findings indeed show that Pt-based Janus TMDs demonstrate the potential for spintronics applications.

## Abstract 2

Stability and electronic properties of 2D Pt-based Janus TMD monolayers with broken centrosymmetry in the crystal structure are demonstrated. The SOC-induced anisotropic Rashba and Dresselhaus splitting is present near the Fermi level.

## Graphical abstract

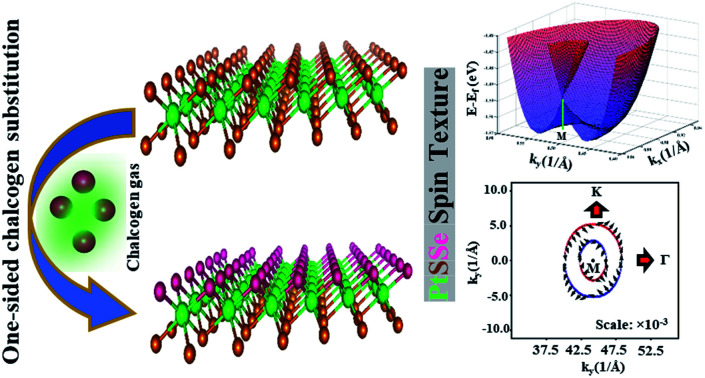

## 1. Introduction

The discovery of exfoliated graphene has been an important milestone in the field of two dimensional (2D) materials.^1^ Over the years, it has been extensively studied and was found to have exemplary properties such as high electron mobility, low resistivity, large breaking strength, and others that have various applications.^2–6^ However, graphene was found to possess a small band gap and very weak spin–orbit coupling (SOC), which are not suitable for semiconductor applications.^4–6^ As a result, research on materials having the same hexagonal structure as graphene, but with different electronic and magnetic properties, has been gaining momentum ever since.^4–24^ Some materials that are currently of interest are MXenes,^7–9^ hexagonal boron nitride,^10^ group IV-based^11^ and group III–V^12^ based honeycombs, transition metal dichalcogenides (TMDs),^13–19^ and Janus TMDs.^20–27^ Of all these materials, Janus 2D TMDs have recently been in the spotlight because of their novel properties^20–27^ due to breaking of symmetry *via* heterostructures,^21–26^ functionalization,^21–24,26^ or substitution.^20–22,24,26,27^

TMDs are defined by a general formula of MX_2_ with M corresponding to the transition metal, and X corresponding to chalcogens S, Se, or Te. On the other hand, Janus 2D TMDs are materials derived from the original TMD monolayer with the chemical formula of MXY, where one of the X is replaced by a different element (Y) such that Y can be a chalcogen, halogen, or pnictogen.^20^ Due to the difference in elements, Janus TMDs possess non-uniform charge distribution and symmetry breaking which have led them to manifest unique properties. Recently, monolayer MoSSe has been successfully fabricated by a selenization process from MoS_2_ (ref. 28) and sulfurization process from MoSe_2_,^29^ while the WSSe thin film by chemical vapor deposition,^30^ which has served as an inspiration for theoretical studies with these materials.^31–42^ Moreover, theoretical studies have predicted Janus 2D TMDs to have various applications such as band engineering,^31,32^ sensors,^33^ optoelectronics,^34^ valleytronics,^35^ magnetic memory devices,^36^ transistors^37^ and the Rashba effect.^38–42^

Materials with significant SOC strength are particularly interesting because spin will be included in the system in addition to electron charge. This leads to various novel phenomena that have important applications in spintronics.^42–46^ One of the fascinating phenomena is the Rashba effect,^43,47,48^ which is a momentum-dependent spin splitting in materials with asymmetry.^43,48^ Experimental observation of the Rashba effect is done by magnetotransport measurements, in the case of AlGaAs/GaAs,^49^ and angle resolved photoemission spectroscopy (ARPES) techniques, like in the case of Bi_2_Se_3_ (ref. 50) and PtBi_2_ (ref. 51).Theoretical investigations have predicted that the Rashba effect exists in many 2D materials such as in heterostructures,^52,53^ metal–semiconductors,^54,55^ heavy metal films,^56^ and in Janus 2D TMDs.^21,24,42,57,58^ Interestingly, the effect of Rashba spin splitting can be further tuned^59,60^ by applying external electric fields and by strain engineering, which have exciting applications in spintronics,^61^ Majorana zero modes,^62^ optics,^63^ and magnetism.^64^

Among the Janus 2D TMDs, studies regarding PtXY (X,Y = S, Se, or Te) have been gaining some attention,^65–70^ mostly focusing on their optical,^65^ structural stability,^66^ mechanical,^66,67^ electronic,^67,68^ and thermal properties.^69^ In addition, monolayer Janus PtSSe has already been experimentally realized.^70^ However, most of the studies did not explore further on the effect of breaking of symmetry and SOC, giving rise to Rashba splitting. Thus, we attempt to fill this gap by focusing our attention on the Rashba splitting in PtXY. In this study, we find that the PtXY monolayers retain the 1T phase based on the calculated formation energies, and are also thermodynamically stable based on phonon calculations. In addition, their electronic properties exhibit an insulating phase with indirect band gaps. Moreover, we observe that the anisotropic Rashba splitting occurs in the M–Γ and M–K directions, similar to other Janus 2D TMDs, such as in MoSSe.^58^ However, further analysis of the spin texture shows that PtXY Janus monolayers exhibit a combination of Rashba effect and Dresselhaus-like contribution. Finally, we highlight that the strength of the Rashba effect and band gap can be tuned by applying biaxial strain. These results are promising for future applications in spintronics.

## 2. Methodology

The systematic first-principles calculations on Janus PtXY monolayers were performed within the density functional theory framework as implemented in the Vienna *Ab initio* Simulation Package (VASP)^71^ using the projector-augmented wave (PAW)^72^ and the Perdew–Burke–Ernzerhof (PBE) functional with an energy cut-off set to 600 eV. The volume and atomic positions of the crystal structures were allowed to relax until the residual force acting on each atom was less than 10^−3^ eV Å^−1^, and the self-consistent convergence criterion for electronic structures was set to 10^−6^ eV. The first Brillouin zone (BZ) was sampled using a Γ-centered Monkhorst–Pack^73^ grid of 36 × 36 × 1, and a vacuum of 20.0 Å was added to eliminate the interaction due to periodic boundary conditions. The formation energies were calculated for both monolayer 2H and 1T phases to verify their stable structures. To further investigate the thermodynamic stability of the structures, phonon spectral calculations were performed using Phonopy.^74^ Moreover, hybrid functional approach HSE06 (ref. 75 and 76) calculations were also performed to obtain the accurate band gaps for the unstrained cases. SOC was included in all the self-consistent calculations. These two calculation settings are necessary to replicate and predict experimental results with higher accuracy. Finally, an in-plane biaxial strain from −3.0% to 5.0% was applied and the atomic positions were allowed to relax.

## 3. Results and discussion

**Fig. 1:**
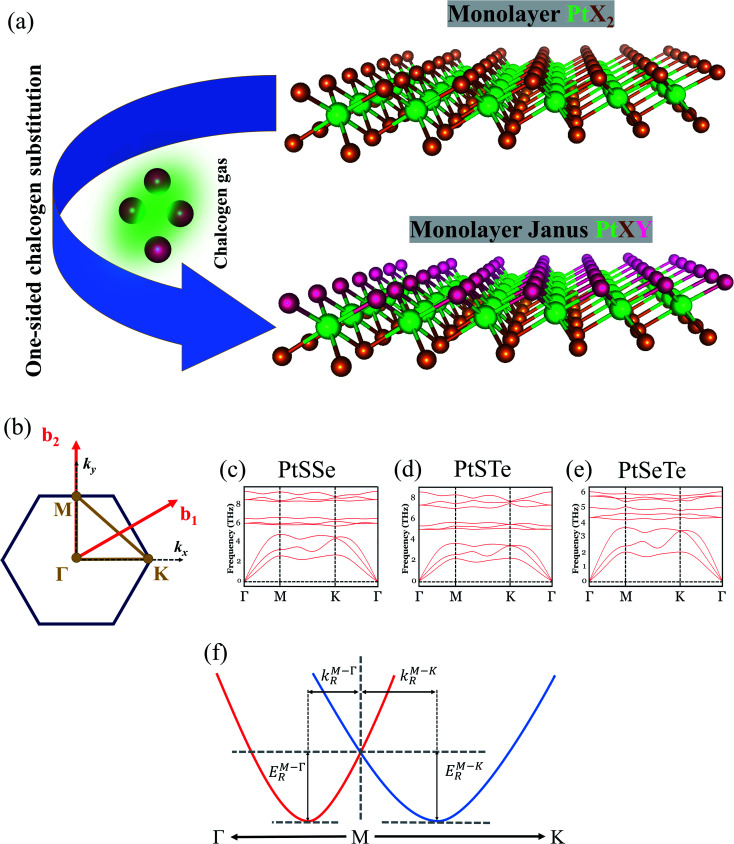
(a) Representation of the fabrication process of monolayer 1T Janus PtXY (X,Y = S, Se, or Te) from the parent PtX_2_ material. (b) 2D Brillouin zone with high symmetry points. The phonon dispersions of (c) PtSSe, (d) PtSTe, and (e) PtSeTe. (f) Schematic diagram of the anisotropic Rashba spin-splitting with energies (*E*^M–Γ^_R_ and *E*^M–K^_R_) and momentum offsets (*k*^M–Γ^_R_ and *k*^M–K^_R_).

In this study, we first establish the preferred crystal structure of the Janus PtXY monolayers, in comparison with their parent materials, PtX_2_ (ref. 14 and 19) TMDs. Fig. 1a shows the schematic diagram of the fabrication process of monolayer 1T Janus PtXY (X,Y = S, Se, or Te) through chalcogen substitution from its parent PtX_2_ material, and Fig. 1b shows the corresponding first BZ. Similar to monolayer TMDs (MX_2_), the monolayer PtXY possesses a graphene-like honeycomb lattice in which the transition metal (Pt) is sandwiched between two different chalcogen atoms. The Pt atoms are bonded to three X and three Y atoms on each side, forming a *C*_3v_ point group. The calculated formation energy per formula unit (eV f.u.^−1^) of the PtX_2_ and PtXY monolayers indicates that the more stable structure is 1T, as compared to 2H, and is summarized in Table 1. Our results are in agreement with recent studies,^66,67^ with minor differences in lattice parameters which originate from the different van der Waals corrections used in the calculations.

**Table 1:** The calculated formation energies per formula unit (eV f.u.^−1^) for pristine PtX_2_ and Janus PtXY monolayers in 1T and 2H structures

Structure	Formation energy per formula unit (eV f.u.^−1^)					
	PtS_2_	PtSe_2_	PtTe_2_	PtSSe	PtSTe	PtSeTe
1T	−2.34	−1.48	−1.07	−1.87	−1.45	−1.22
2H	−0.58	−0.17	−0.24	−0.38	−0.53	−0.19

Additionally, phonon dispersions in Fig. 1c–e reveal that 1T PtXY are thermodynamically stable^53^ and in agreement with recent studies.^66,67,70^ Moreover, the 2D PtX_2_ parent materials have been experimentally synthesized^19,77,78^ in 1T, as well as PtSSe,^70^ hence implying the stability of the resulting PtXY in 1T structure. Thus, we focus our attention on the 1T phase for the rest of the discussion. The corresponding optimized lattice constants of PtXY in the 1T phase are listed in Table 2. Here, we see the trend that the lattice constants gradually increase as the sum of the atomic radii of X and Y chalcogen atoms increases. Further, due to the different elements, the bond lengths of Pt–X and Pt–Y are different (see Table 2), and thus the centrosymmetry is broken.

**Table 2:** Structural parameters *a* (lattice constant in Å) and *d*_TM_ (the bond length in Å), bandgap *E*_HSE06_ and *E*_PBE_ with SOC using HSE06 and PBE calculations (eV), respectively, and *α*^M–X^_R_ is the Rashba parameter (eV Å^−1^) where X is Γ or K for M–Γ and M–K directions, respectively. The corresponding effective masses (*m*_e_) of each material are also provided

Material	*a* (Å)	*d*_TM-S_ (Å)	*d*_TM-Se_ (Å)	*d*_TM-Te_ (Å)	*E*_HSE06_ (*E*_PBE_) (eV)	Effective mass (*m*_e_)		*α* ^M–Γ^_R_ (eV Å^−1^)	*α* ^M–K^_R_ (eV Å^−1^)
						M–Γ	M–K		
PtSSe	3.659	2.431	2.500	—	2.108 (1.520)	0.091	0.136	1.654	1.333
PtSTe	3.807	2.497	—	2.625	1.335 (0.930)	0.136	0.272	1.103	1.244
PtSeTe	3.892	—	2.590	2.650	1.221 (1.050)	0.068	0.068	0.435	0.746

**Fig. 2:**
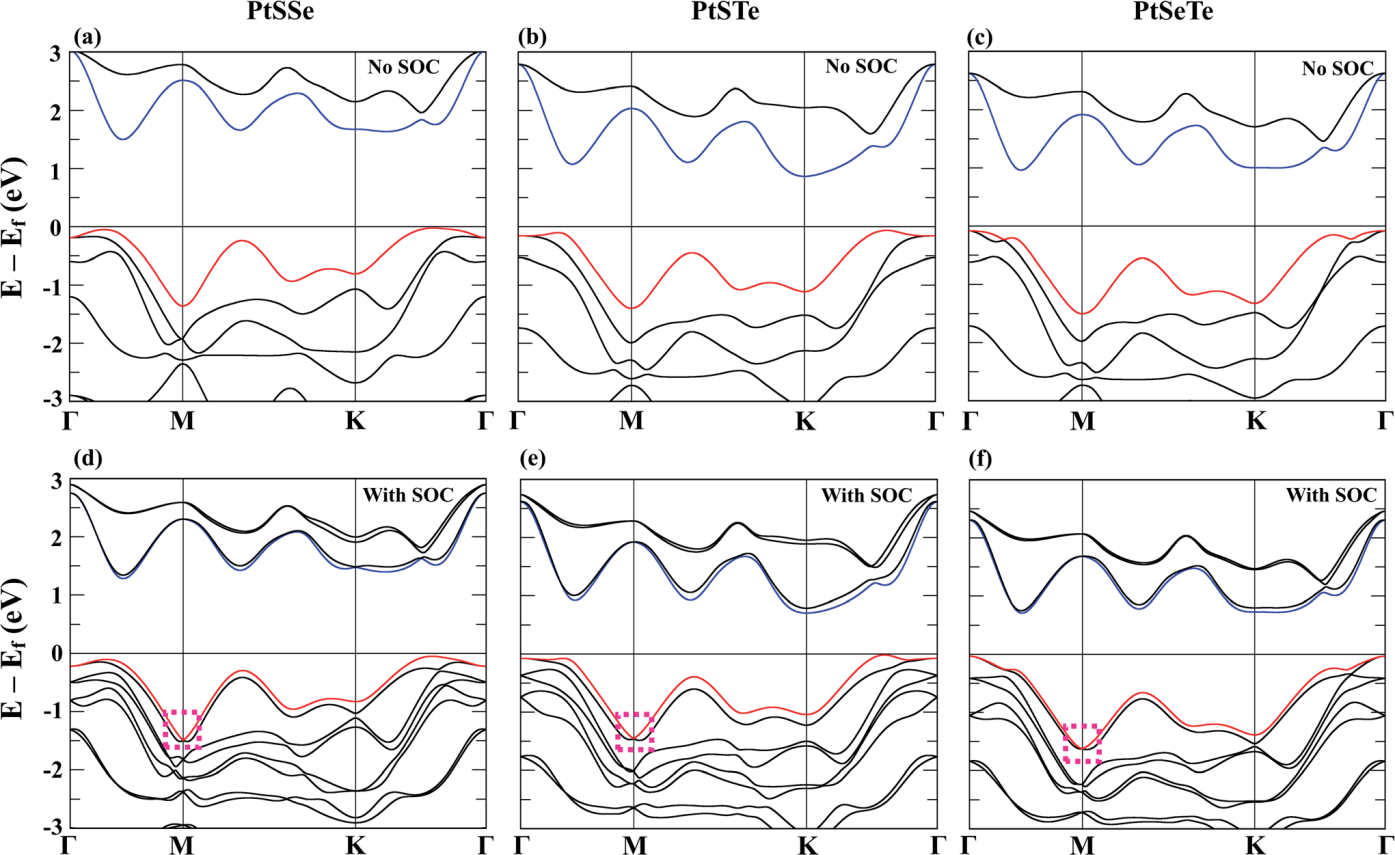
Band structures of the PtSSe monolayer (a) without and (d) with SOC, PtSTe monolayer (b) without and (e) with SOC, and PtSeTe monolayer (c) without and (f) with SOC using PBE functional. The red and blue lines correspond to the VBM and CBM, respectively. The dashed magenta square highlights the anisotropic Rashba effect of PtXY Janus monolayers.

After confirming the stability, we next discuss the electronic properties of PtXY. All the Pt Janus monolayers considered in this study exhibit an insulating phase, as shown in the band structures in Fig. 2a–c and d–f for without and with SOC, respectively. For all the materials, the lowest point of the conduction band minimum (CBM) is located in-between Γ and M, while the highest point of the valence band maximum (VBM) is located in-between K and Γ for PtSSe and PtSTe, and at Γ for PtSeTe. The corresponding calculated band gaps using PBE and HSE06 with SOC are shown in Table 2. Using the HSE06 (PBE) functional, the PtSSe monolayer exhibits the largest indirect band gap of 2.108 eV (1.520 eV), and then PtSTe and PtSeTe exhibit indirect band gaps of 1.335 eV (0.930 eV) and 1.221 eV (1.050 eV), respectively. Our DFT-PBE results predict that PtXY are indirect band gap semiconductor, similar to recent studies.^66,67^ Furthermore, in order to obtain accurate band gaps, HSE06 calculations were included in our study, as shown in Table 2. We note that HSE06 calculations were not included in the previous two studies.^66,67^

We now examine in more detail the monolayer band structures upon the inclusion of SOC. As shown in Fig. 2d–f, anisotropic Rashba splitting can be observed at the VBM around the M point when SOC is included, as highlighted by the dashed magenta square. The Rashba splitting occurred due to the potential gradient generated by the asymmetric distribution of charges.^79^

For 2D systems, the Bychkov–Rashba Hamiltonian can be written as^38,80,81^1
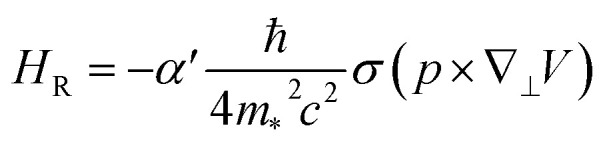
where *α*′ is the Rashba primary correlation factor, *ħ* is the reduced Planck constant, m_*_ is the effective mass, *c* is the speed of light in vacuum, *σ* is the Pauli spin matrices, *p* is the linear momentum defining the momentum space, ∇_⊥_ is the gradient operator, and *V* is the electric potential. Incorporating the perpendicular electric field (*E*_z_) in eqn (1), we get the following expression:^38^2
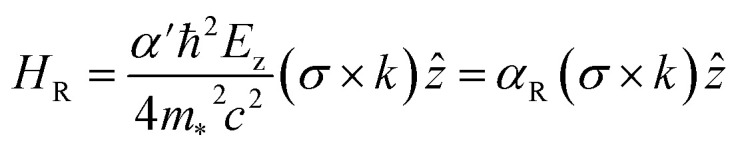
where *k* represents a vector in momentum space.

From eqn (2), the general Rashba interaction coefficient, commonly referred to as the Rashba parameter *α*_R_, is given by:^38^3
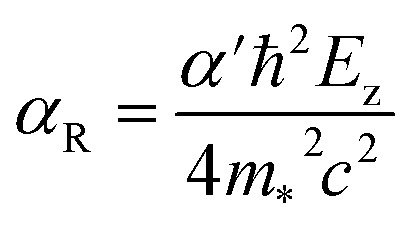


Hence, the dispersion relation for the Rashba spin-splitting phenomenon is:^38,81,82^4
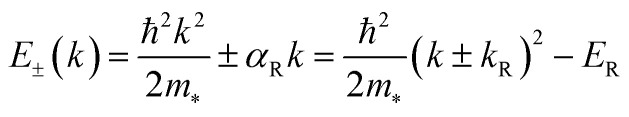


This relation describes two parabolas that are shifted from the origin. We can then derive from eqn (4) the expression for the Rashba parameter *α*_R_ in terms of the Rashba energy *E*_R_ and momentum offset *k*_R_ from the *k* vector:^38,83^5
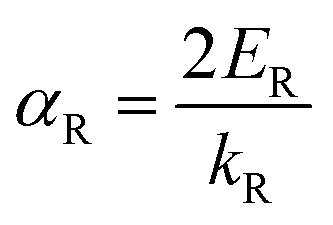


For systems with anisotropic Rashba spin-splitting (see Fig. 1f), the Rashba parameter defined in eqn (5) can be further modified to incorporate the different directions of *k*_R_, such that:6
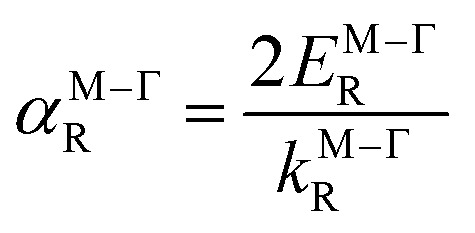
7
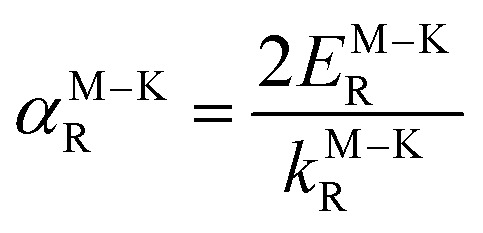
where eqn (6) and (7) correspond to the Rashba parameter *α*_R_ along the M–Γ and M–K directions, respectively. The calculated *α*^M–Γ^_R_ and *α*^M–K^_R_ for PtXY monolayers with their corresponding effective masses (m_*_) are summarized in Table 2. The calculated values of *α*^M–Γ^_R_ are 1.654, 1.103, and 0.435 eV Å^−1^, while the calculated values of *α*^M–K^_R_ are 1.333, 1.244, and 0.746 eV Å^−1^, for PtSSe, PtSTe, and PtSeTe, respectively.

It is clearly evident that the Rashba splitting is indeed anisotropic and is highly dependent on the directions selected in the Brillouin zone. Moreover, both *α*^M–Γ^_R_ and *α*^M–K^_R_ decrease as the net atomic mass, hence the SOC strength, of the XY chalcogens increases (SSe < STe < SeTe). Furthermore, we would like to note that Janus MoXY and WXY TMD^58,84^ monolayers retain the 2H phase (trigonal), while in our study, PtXY retains the 1T phase (octahedral). Because of their intrinsic crystal symmetry, breaking of mirror symmetry occurs in MoXY and WXY, while the breaking of inversion symmetry occurs in PtXY. Another difference between MoXY (or WXY) and PtXY Janus monolayers is that the Rashba splitting for MoXY (or WXY) occurs at Γ, while for PtXY it occurs at M. A comparison of the Rashba parameter of PtXY monolayers to the well-known Rashba materials is shown in the ESI Table S1.†

**Fig. 3:**
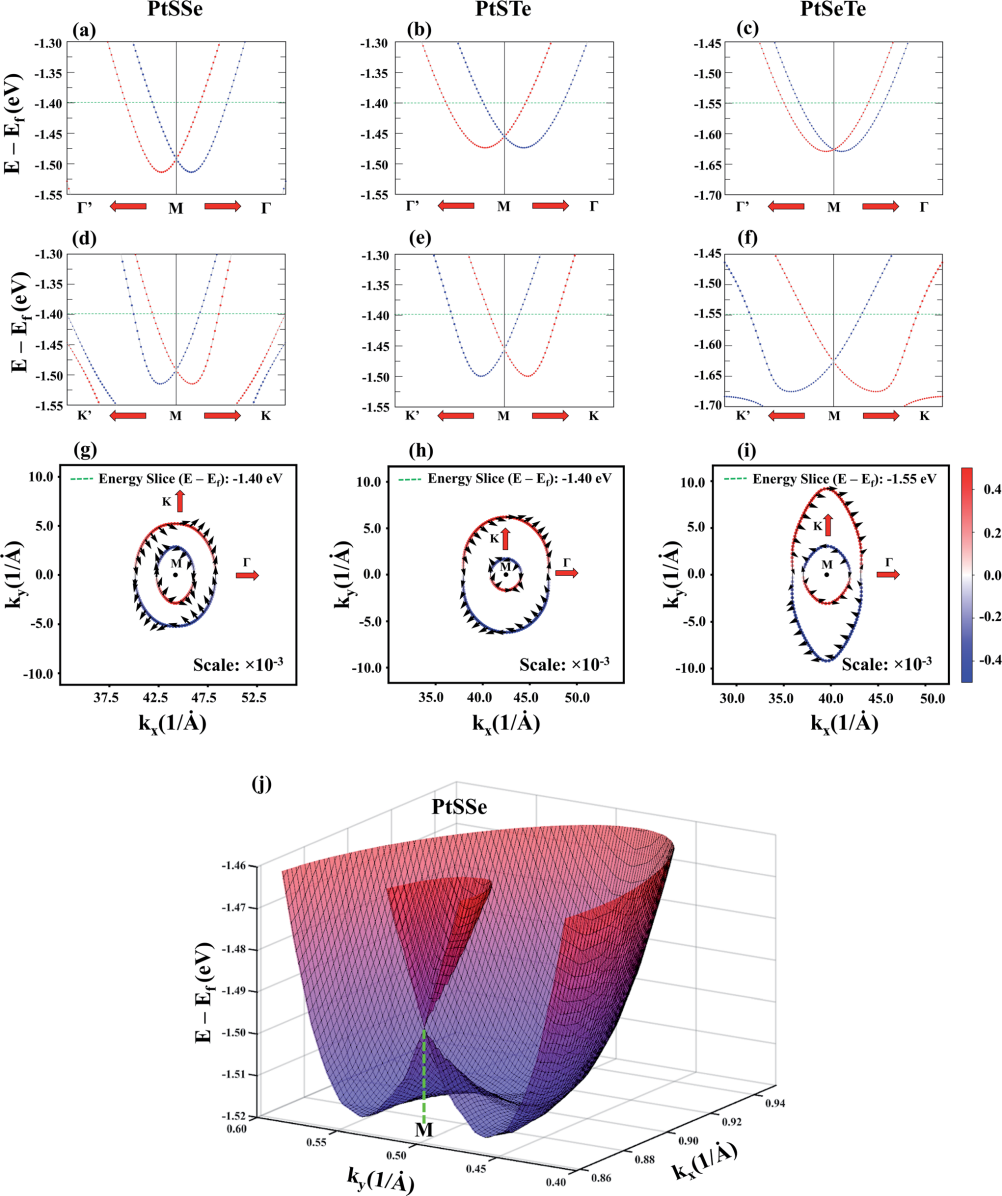
The band structures using the PBE functional with SOC of (a and d) PtSSe, (b and e) PtSTe, and (c and f) PtSeTe along the (a–c) Γ′–M–Γ and (d–f) K′–M–K *k*-path directions. The red and blue circles of (a–c) correspond to *S*^+^_*x*_ and *S*^−^_*x*_, respectively. The red and blue circles of (d–f) correspond to *S*^+^_*y*_ and *S*^−^_*y*_, respectively. The dashed green line corresponds to the energy slice of the 2D spin texture. The energy contour of the 2D Fermi surface spin-textures of (g) PtSSe at *E* − *E*_F_ = −1.3 eV, (h) PtSTe at *E*−*E*_F_ = −1.35 eV, and (i) PtSeTe at *E* − *E*_F_ = −1.55 eV. The direction of the arrows corresponds to *S*_*x*_ and *S*_*y*_, while the red and blue colors correspond to *S*_*z*_. (j) 3D band structure of PtSSe. The color gradient corresponds to the energy along the *z*-axis. The two parabolas crossing at the M-point are the evidence of the Rashba effect.

To further elucidate the mechanism of the Rashba spin splitting in PtXY monolayers, we show in Fig. 3a–c and d–f the magnified images of the band splitting at the VBM along the Γ′–M–Γ and K′–M–K directions, respectively. The significant difference in the magnitude and shape of the band splitting due to the Rashba effect emphasizes the anisotropic Rashba spin splitting exhibited by PtXY. In addition to the band structure, the spin-resolved constant energy 2D spin textures are also plotted in Fig. 3g–i. The black arrows represent the in-plane *S*_*x*_ and *S*_*y*_ vector components, while the blue-to-red color gradient represents the out-of-plane *S*_*z*_ vector component. It is clearly shown that the spin arrows in the outer band exhibit a clockwise rotation, while the spin arrows in the inner band exhibit a counterclockwise rotation, thus visually confirming the characteristic signature of the Rashba splitting. Also, the elliptical shapes of the inner and outer branches of the split band also highlight the anisotropic band splitting. For visual representation, the 3D band structure of PtSSe is shown in Fig. 3f. The two parabolas crossing at the M-point emphasize the Rashba effect. Interestingly, it is also evident in the spin textures that the orientation of the spin directions is not purely rotational, which is in contrast with the characteristic feature of pure Rashba-induced spin splitting.^85^ To explain this peculiar phenomenon, it is important to note the two types of SOC splitting with inversion asymmetry, namely Rashba^48,80^ and Dresselhaus^47^ effects. For the pure Rashba SOC, the spin is always orthogonal to the *k* vector, while for the pure Dresselhaus SOC, the spin can be either parallel or orthogonal to the *k* vector.^85^ In the case of PtXY monolayers, it is obvious that the spin-splitting is primarily due to the Rashba effect, and the notable presence of a Dresselhaus-like contribution plays a secondary role in the observed phenomenon.^86,87^ In this scenario, we highlight that the Dresselhaus effect is caused by the bulk inversion asymmetry (BIA).^87^ The parent PtX_2_ monolayers in the 1T structure possess the inversion symmetry, but upon transition to the Janus PtXY monolayer structure, the breaking of this symmetry occurs. This results in the Dresselhaus effect in the 2D thin film, in addition to the Rashba effect.

We further note that for materials with spin splitting at the gamma point, *e.g.* W-based and Mo-based materials, the major contributing factor is purely due to the Rashba effect. In contrast, for materials such as PtXY monolayers where the spin splitting occurred at the low symmetry M-point, the interplay between the Rashba and Dresselhaus effects is the crucial factor. The potential applications of materials with these exotic properties are persistent spin helix observed in magnetic insulators,^88^ GaAs low-dimensional systems,^89,90^ long spin relaxation times,^91,92^ and spin field-effect-transistors^93–95^ for spintronic devices as well as optoelectronic devices.^96^ Moreover, the phenomenon of the Rashba and Dresselhaus effects occurring in 2D systems has not been fully explored, and thus our study provides significant insights into this new type of material.

**Fig. 4:**
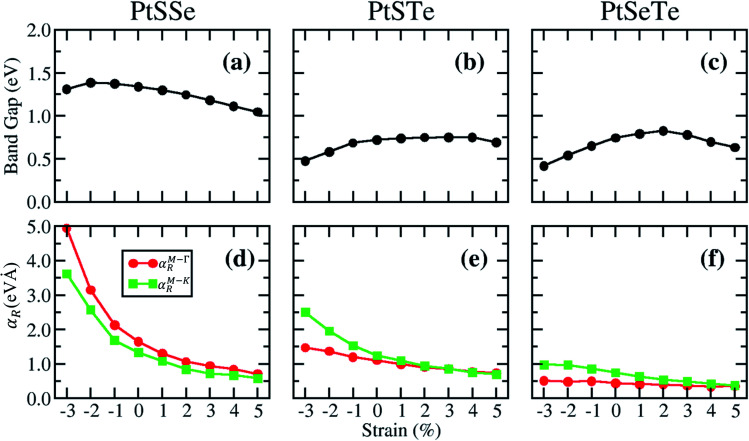
(a–c) Calculated system band gaps using the PBE functional as a function of in-plane biaxial strain for PtXY monolayers when SOC is included. (d–f) Calculated Rashba parameters *α*^M–Γ^_R_ and *α*^M–K^_R_ as a function of in-plane biaxial strain for PtXY monolayers along the M–Γ and M–K directions, respectively.

To explore the manipulation of the Rashba spin splitting in Janus PtXY monolayers, an in-plane biaxial strain is applied in our calculations. The electronic properties of each monolayer under strain (from −3.0% to 5.0%) were studied, and the corresponding band structures are shown in the ESI Fig. S1–S3.† The band gap evolution as a function of strain is plotted in Fig. 4a–c. For the PtSSe monolayer, tensile strain decreases the band gap, while compressive strain increases it until a critical strain of −2.0% where the band gap reaches its maximum value, and then starts to decrease. This trend in bandgap modulation under strain is in good agreement with the recent work.^67^ In contrast, both PtSTe and PtSeTe monolayers experience a decrease in the band gap with compressive strain, while tensile strain increases the band gap until a critical strain of 4.0% and 2.0%, respectively, where the maximum band gaps are reached, and then starts to decrease again. Also, no change in band-gap types, *i.e.* indirect-to-direct or direct-to-indirect transition, was observed upon applying biaxial strain, indicating that Pt-based Janus TMDs preserved some electronic properties of their parent materials.^13,18^ The calculated Rashba parameters *α*^M–Γ^_R_ and *α*^M–K^_R_ as a function of biaxial strain are also presented in Fig. 4d–f. We found that the tensile strain suppresses the Rashba splitting, while the compressive strain enhances it. However, a significant enhancement was only found in PtSSe and PtSTe, while minimal changes were observed in PtSeTe. In general, the Rashba parameters could be further manipulated upon application of compressive strain due to the enhancement of the structural asymmetry of the material. Engineering the material properties such that they will possess a large Rashba parameter is desirable for applications such as spin-field effect transistors^97^ and detection of Majorana fermions.^98^

## 4. Conclusion

We have presented a systematic study of Janus PtXY (X,Y = S, Se, or Te) monolayers using first principles calculations. Formation energy and phonon spectral calculations demonstrate that all the Janus structures are thermodynamically stable in the 1T phase. All the monolayers exhibit an insulating phase with indirect band gaps. Moreover, the Janus PtXY monolayers intrinsically possess anisotropic Rashba-type spin splitting due to the out-of-plane centrosymmetry breaking and the SOC effect. In addition, the different magnitudes of the splitting quantified by the Rashba parameters *α*^M–Γ^_R_ and *α*^M–K^_R_ as determined by the SOC strength of their constituent elements are also presented. Interestingly, the spin texture revealed that the spin-splitting is mainly due to the Rashba effect, but a Dresselhaus-like contribution is also exhibited due to the breaking of inversion symmetry. Finally, the band gap and strength of the Rashba effect can be tuned by strain engineering. With the research development in the SOC-induced Rashba spin splitting of inversion asymmetric systems, broad topics in physics and materials science are now converging to the new age of spintronics. Our results demonstrate the potential of Janus PtXY monolayers in this exciting new field.

## Author contributions

P. A. L. S., L.-Y. F., R. A. B. V., H. N. C., Z.-Q. H., and C.-H. H. performed the data curation, formal analysis, and investigation of the study. F.-C. C. performed the conceptualization, project administration, and supervision of the study. All the authors contributed to the writing of the manuscript, as well as the revisions and editing.

## Conflicts of interest

The authors declare no competing financial or non-financial interests.

## Supplementary Material

NA-003-D1NA00334H-s001
