# Supplementary material for: Anisotropic Rashba splitting in Pt-based Janus monolayers PtXY (X,Y = S, Se, or Te)
Source: Nanoscale Adv. 2021 Sep 14;3(23):6608–16. doi: 10.1039/d1na00334h (PMC9419079; doi:10.1039/d1na00334h)
Supplement: NA-003-D1NA00334H-s001 [file NA-003-D1NA00334H-s001.pdf]

Electronic Supplementary Material (ESI) for Nanoscale Advances.
This journal is © The Royal Society of Chemistry 2021

Supporting Information for Anisotropic Rashba splitting in Pt-based Janus monolayers PtXY (X,Y = S, Se, or Te)

Paul Albert L. Sino[1], Liang-Ying Feng[1], Rovi Angelo B. Villaos[1], Harvey N. Cruzado[1,2], Zhi-Quan Huang[1], Chia-Hsiu Hsu[1], and Feng-Chuan Chuang[1,3,4]*

[1]*Department of Physics, National Sun Yat-sen University, Kaohsiung, 80424 Taiwan*

[2]*Institute of Mathematical Sciences and Physics, College of Arts and Sciences, University of the Philippines Los Baños, College, Laguna, 4031 Philippines*

[3]*Department of Physics, National Tsing Hua University, Hsinchu, 30013 Taiwan*

[4]*Physics Division, National Center for Theoretical Sciences, Taipei, 10617 Taiwan*

[*]Corresponding Author: Feng-Chuan Chuang

Postal Address: 70 Lienhai Rd., Kaohsiung 80424, Taiwan.

Telephone: +886-7-5253733
E-mail Address: fchuang@mail.nsysu.edu.tw

Table S1. Comparison of the Rashba parameter of Janus PtXY monolayers with well-known Rashba materials.

Material	Type	α (eV・Å)	Type of Splitting	Reference
MoS_2/Bi (111)	Heterostructure	1.05	Rashba	[1]
Pb Thin Film	Metal Thin Film	0.04	Rashba	[2]
$PtBi_2$	Bulk	4.36	Rashba + Dresselhaus	[3]
MoSSe	ML Janus TMD	2.0×10^{-3}-1.2	Rashba	[3-5]
WSSe	ML Janus TMD	<1.0	Rashba	[3],[4],[6]
PtSSe	ML Janus TMD	1.65^{*}, $1.33^{\dagger}$	Rashba + Dresselhaus	This Work
PtSTe	ML Janus TMD	1.10^{*}, $1.24^{\dagger}$	Rashba + Dresselhaus	
PtSeTe	ML Janus TMD	0.44^{*}, $0.75^{\dagger}$	Rashba + Dresselhaus	

*α at M-Γ direction, †α at M-K direction

1. K. Lee, W. S. Yun and J. D. Lee, *Phys. Rev. B*, 2015, **91**, 125420.
2. J. H. Dil, F. Meier, J. Lobo-Checa, L. Patthey, G. Bihlmayer, and J. Osterwalder, *Phys. Rev. Lett.*, 2008, **101**, 266802.
3. J. Chen, K. Wu, H. Ma, W. Hu and J. Yang, *RSC Advances*, 2020, **10**, 6388–6394.
4. Y. C. Cheng, Z. Y. Zhu, M. Tahir and U. Schwingenschlögl, *EPL*, 2013, **102**, 57001.
5. F. Li, W. Wei, H. Wang, B. Huang, Y. Dai and T. Jacob, *J. Phys. Chem. Lett.*, 2019, **10**, 559–565.
6. W. Zhou, J. Chen, Z. Yang, J. Liu and F. Ouyang, *Phys. Rev. B*, 2019, **99**, 075160.

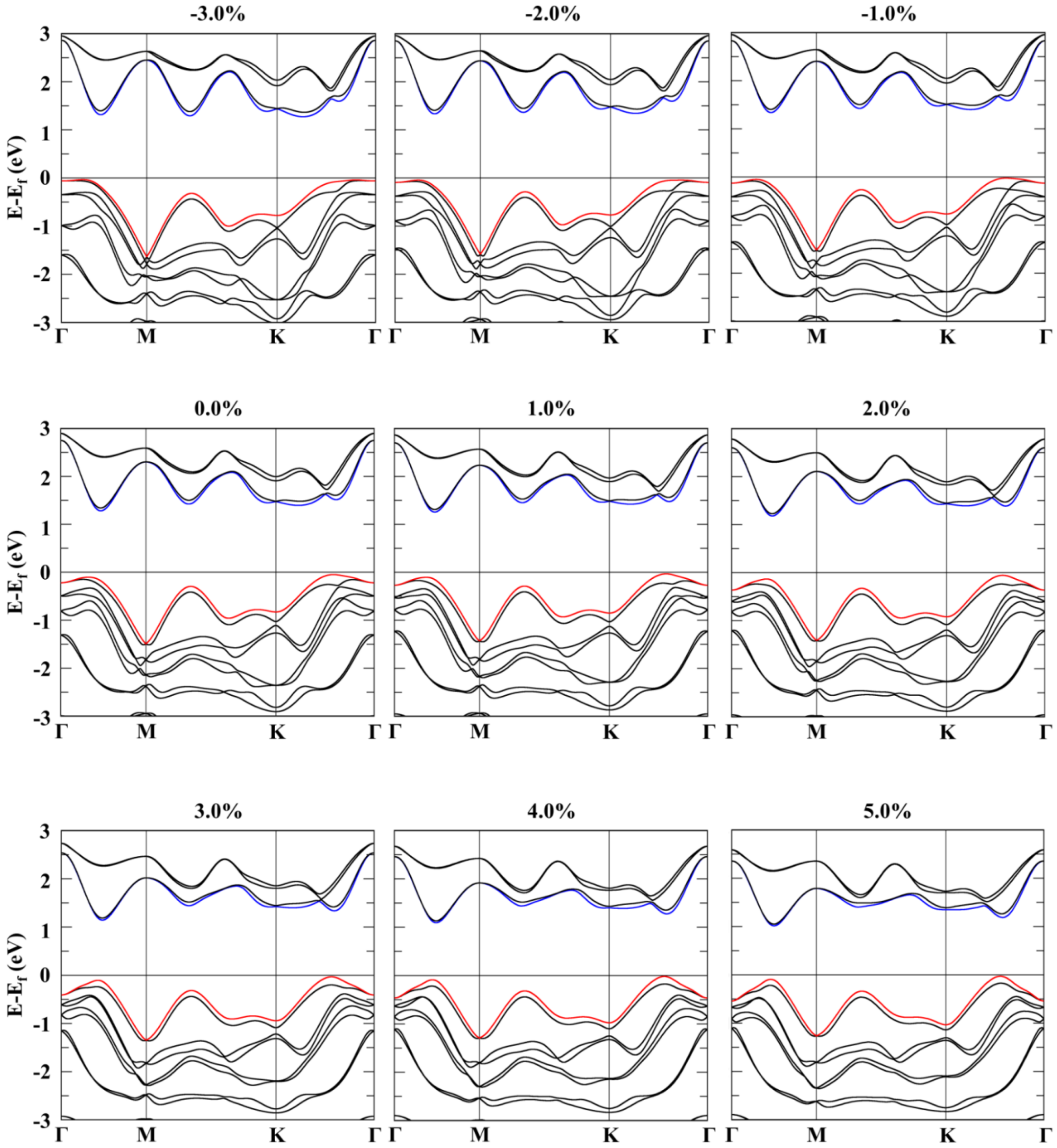

Figure S1. Band structures under spin-orbit coupling (SOC) of monolayer Janus PtSSe under varying strain from -3.0% to 5.0%. The red and blue colored bands correspond to the valence band maximum (VBM) and conduction band minimum (CBM).

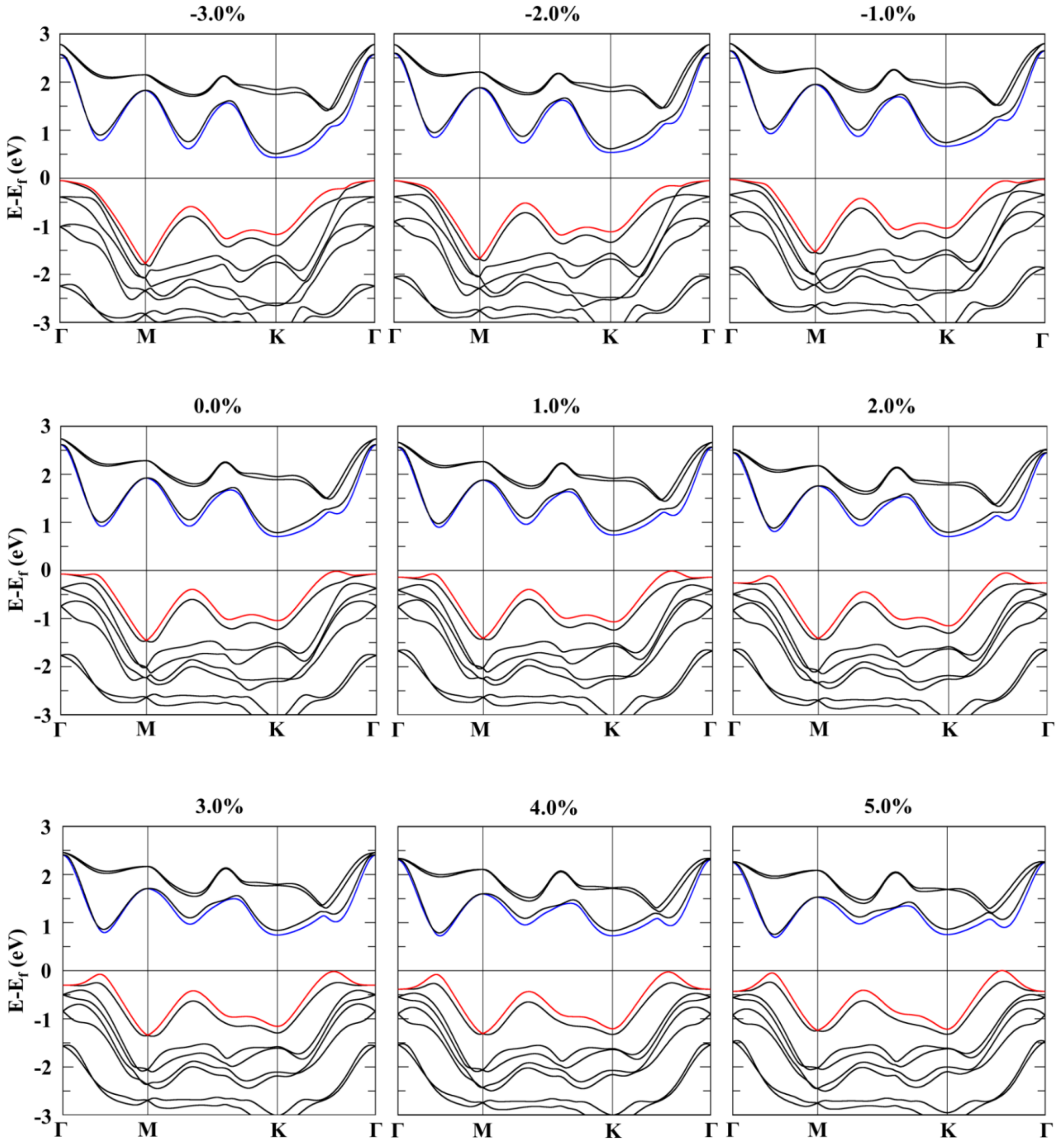

Figure S2. Band structures under SOC of monolayer Janus PtSTe under varying strain from -3.0% to 5.0%. The red and blue colored bands correspond to the VBM and CBM.

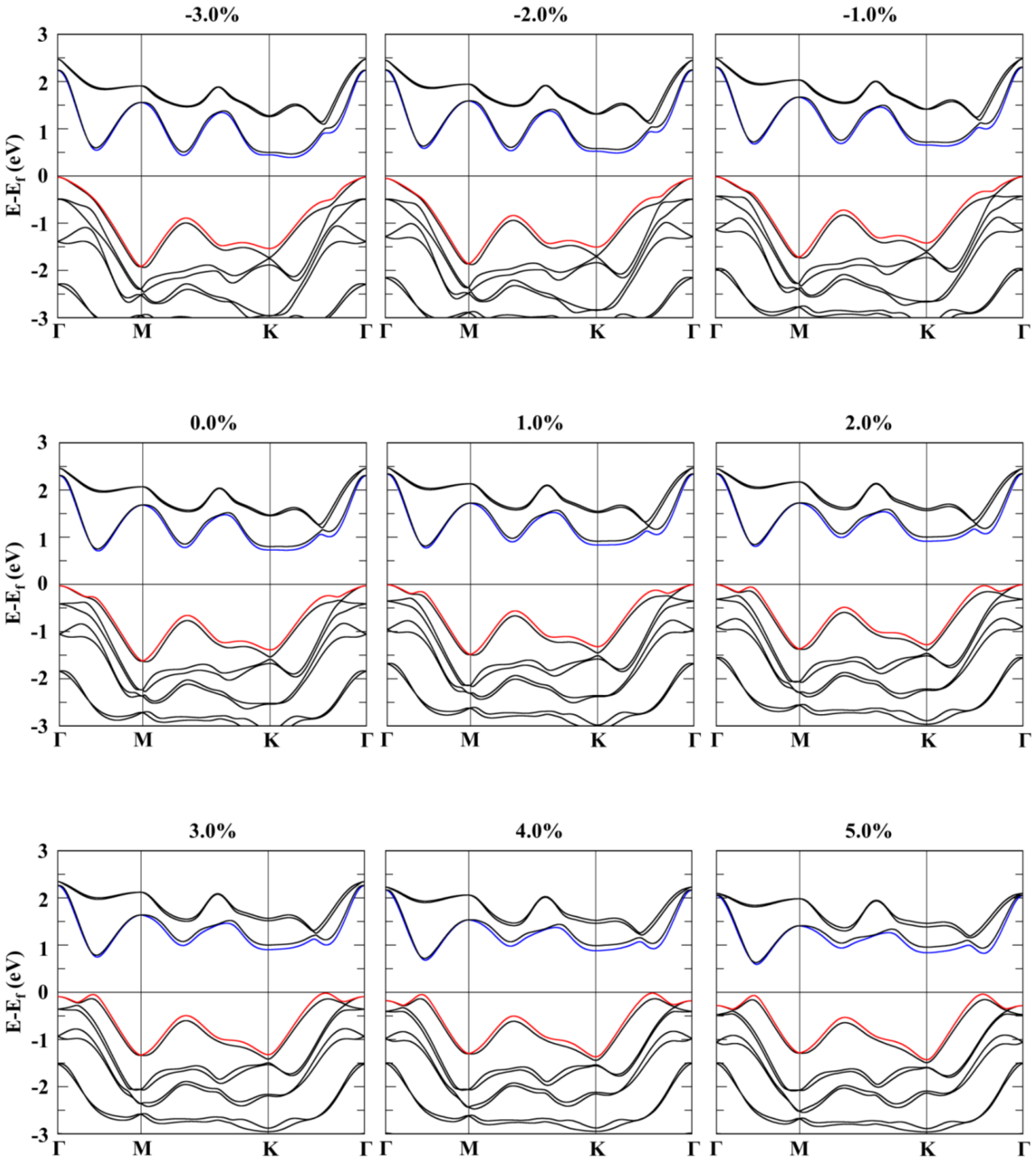

Figure S3. Band structures under SOC of monolayer Janus PtSeTe under varying strain from -3.0% to 5.0%. The red and blue colored bands correspond to the VBM and CBM.
